# Puffery

**Published:** 1871-03

**Authors:** 


### PUFFERY.

Hardly one dental journal but stands self-convicted of puffery. The term requires no explanation. In these days of cheap newspapers and universal printing, it is perfectly understood.

Is it a new invention or an improved material? Forthwith the periodicals gush in its praise, and by that sort of "come along John," which carries the crowd, every operator goes and buys. The purchase may be a fraud, or what is almost as bad, an impracticable thing, but what of that? It was commended by the dental journals, and that was enough.

The design here is not to depreciate the legitimate efforts of an inventor or manufacturer, to bring the result of his skill and industry before the world, but to show that puffery will injure more than benefit anything whether good or bad in itself. If it is good, and that can only be ascertained by the test of experience, it will work its way to the surface; if bad, puffery will not save it from condemnation, and only bring legitimate advertising into bad odor.

The specific object of these paragraphs, is to bring to the judgment of intelligent readers the wrong of puffery, whether in print or in the proceedings of societies. We submit that the appliances of every profession are not important—are hardly proper subjects for discussion; it is the ability, the experience, and the methods which they execute that should engage critical attention, and no publisher who doses his readers with recommendations of this man's instruments and that man's materials, or who even interlards the common intelligence of the profession with them, is doing his duty as a publisher.

The best newspapers will not admit a line to their columns of a puffy character. For all that sort of thing there is a department at so much per square, where every patentee, or inventor, or vendor can blow his own horn, none daring to make him afraid. And this puts the thing where it properly belongs, for when motives come to be weighed, the common sense of the majority of readers will be wondering how much the writer got for his handsome notice of Mr. So and So's new instrument, feeling quite satisfied that it was too new to have given witness of its own excellence, without that questionable recommendation. B.

